# Feasibility of Using Multilayer Platelets as Toughening Agents

**DOI:** 10.3390/ma3010001

**Published:** 2009-12-24

**Authors:** Yuan-Liang Chin, Wei-Hsing Tuan

**Affiliations:** Ceramic-Matrix Composite Lab., Dept. of Mater. Sci. & Eng., National Taiwan University, Taipei, 106 Taiwan; E-Mail: xdollar@mail.mse.ncku.edu.tw (Y.-L.C.)

**Keywords:** composite, toughening, platelet, crack deflection

## Abstract

It is known that the toughness of brittle ceramics can be improved significantly with the addition of hard platelets. In the present study, platelet-shape multilayer ceramic laminates are utilized as a toughening agent for alumina ceramics. They are prepared by laminating the BaTiO_3_-based ceramic tapes. Although the elastic modulus of the BaTiO_3_-based platelets is lower than that of the alumina matrix, and the platelets are also reactive to alumina at elevated temperatures, the weak platelets are found to exhibit the ability to deflect major matrix cracks by forming a large number of microcrack branches within the platelets, thus achieving the desired toughening effect.

## 1. Introduction

The brittle nature of ceramics imposes limits on their applications as structural components. To improve the toughness of ceramics is therefore a long-standing pursuit for many ceramists. The approach of adding ceramic whiskers or platelets has attracted a lot of attentions. The toughness of alumina could be enhanced by two times after the addition of SiC whiskers [1]. However, the health issue associated with the whiskers has prohibited their further usage. Ceramic platelets were proposed as an alternative to replace ceramic whiskers. Since the size of platelets is relatively large (10~100 μm in diameter and 1~10 μm in thickness); there is no health concern associated with the use of such ceramic platelets. Several reports had demonstrated that the toughness of a ceramic matrix could be improved by 10 to 50% after the addition SiC platelets [2,3,4]. However, the strength of the platelet-toughened ceramics is relatively lower than that of matrix alone. Furthermore, due to the anisotropic shape of platelets, microstructure anisotropy as well as the toughness anisotropy were observed for the platelet-toughened composites [3]. The major hurdle of using ceramic platelets is their sources are very limited. Furthermore, the cost of the SiC and Al_2_O_3_ platelets is relatively high. 

**Figure 1:**
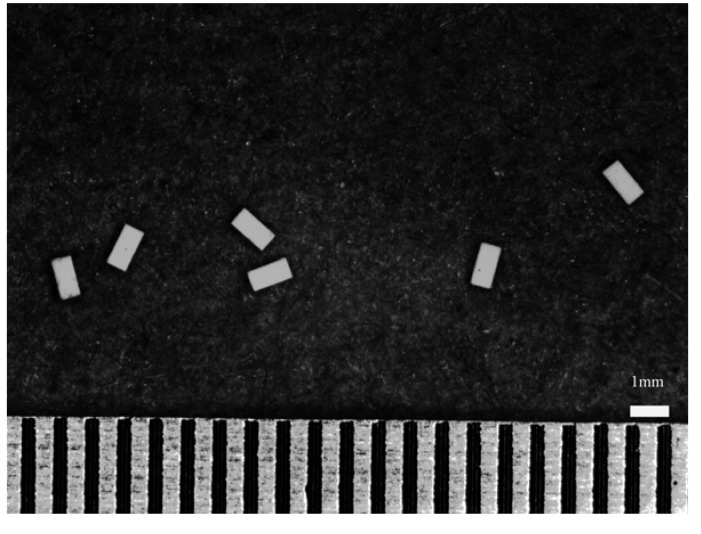
Morphology of the platelets used in the present study.

Many passive components, such as ceramic capacitors, are in the shape of platelets. A typical morphology of ceramic capacitors is shown in Figure 1. Due to the demands for miniaturization, nowadays ceramic capacitors are usually manufactured by employing multilayer technology [5,6]. The size of the multilayer components has decreased from ~3.2 × 1.6 × 1.5 mm to ~0.6 × 0.3 × 0.3 mm in the last decade. Even smaller components, such as ~0.3 × 0.2 × 0.2 mm components are now available, and this downsizing trend shows no signs of slowing down, showing that the processing technology for manufacturing multilayer components is moving forward. Furthermore, the price of multilayer components is also decreasing. These multilayer components are in the shape of platelets, so it was therefore of interest to investigate the feasibility of using multilayer components as ceramic toughening agents. During the manufacturing of multilayer components, the edge of the tape is not printed with electrode paste. These dummy components, the components without inner electrodes, are produced along with the other multilayer components. Since there is no expensive inner electrode present, the cost of these dummy components is expected to be even more competitive. In the present study, the feasibility of using dummy components as the toughening agent is evaluated. 

## 2. Results and Discussion 

**Figure 2:**
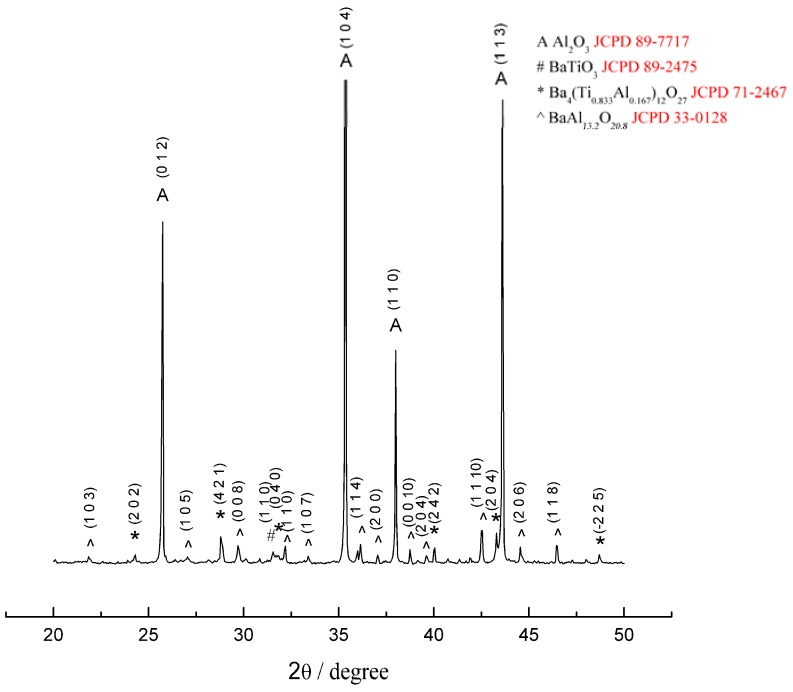
XRD pattern of Al_2_O_3_/14wt%BaTiO_3_-platelet composite after hot-pressing at 1,400 °C.

**Figure 3:**
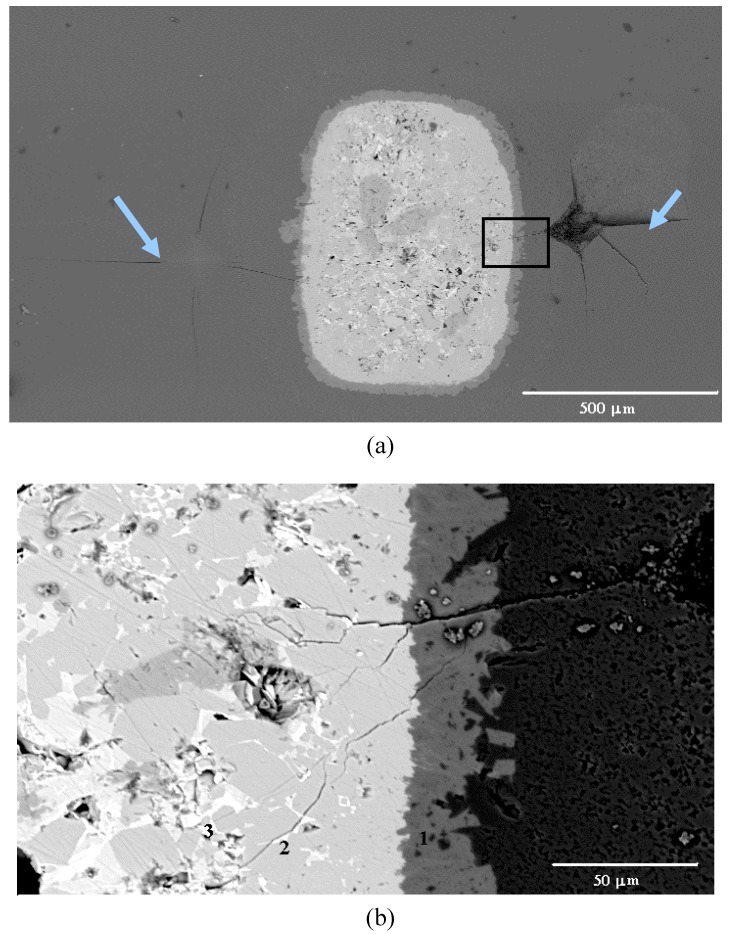
(a) A typical BaTiO_3_-platelet in the Al_2_O_3_/BaTiO_3_-platelet composite. Two indentations are arrowed. (b) Interactions between an indentation-induced crack and platelet.

Figure 1 shows the morphology of the green dummy platelets used in the present study. Figure 2 shows the XRD pattern of the Al_2_O_3_/BaTiO_3_-platelet composite after hot-pressing at 1,400 °C for 1 h. Apart from Al_2_O_3_ and a small amount of BaTiO_3_, two reaction phases, Ba_4_(Ti_0.833_Al_0.167_)_12_O_27_ and BaAl_13.2_O_20.8_ are found. A typical platelet within the Al_2_O_3_ matrix after hot pressing is shown in Figure 3(a). A reaction layer is present between Al_2_O_3_ matrix and BaTiO_3_ platelet. Within the platelet, there are two phases: a white phase and a gray phase (Figure 3b). The energy-dispersive X-ray (EDX) analysis was conducted to determine the composition of each phase. By combining the XRD and EDX results, the reaction layer (denoted with 1 in Figure 3b) between matrix and platelet is a BaAl_13.2_O_20.8_ phase. The gray phase within the platelet is a Ba_4_(Ti_0.833_Al_0.167_)_12_O_27_ phase (denoted with 2 in Figure 3b), the white one is the residual BaTiO_3_ phase (denoted with 3 in Figure 3b). The amount of BaTiO_3_ phase is the lowest in the composites. 

Typical SEM micrographs to demonstrate the interactions between crack and platelet are shown in Figure 3. Two Vickers indentations are introduced near the platelet, Figure 3(a). The indentation at the left-hand side of the platelet induces four cracks at each indent corner. One crack propagates straight into the platelet then disappears within the platelet. The indentation at the right-hand side of the platelet is much closer to the platelet. This indentation also produces four major cracks in the matrix. One major crack penetrates into the interfacial reaction layer, then forms many small crack branches within the platelet, Figure 3(b). 

Table 1 shows the characteristics of each phase within the composites. These values are determined by using the nano-indentation technique at a very small load of 50 mN. No crack was observed at the indent with the optical microscope after the nano-indentation, though micro-cracks may still be formed under the surface [7]. This technique allows us to determine the *in situ* physical characteristics of each phase in the composite. The measured elastic modulus of Al_2_O_3_ and BaTiO_3_ is 411 GPa and 180 GPa, respectively. The measured value for Al_2_O_3_ is close to the values reported previously [8]. The measured elastic modulus of BaTiO_3_ is 180 GPa, which is higher than the reported values for barium titanate (107~120 GPa) [9]. It may be partly due to that the BaTiO_3_ phase is surrounded by the rigid Al_2_O_3_ matrix. It may also be related to the solution of some Al ions into BaTiO_3_ grain. 

**Table 1:** Elastic modulus, hardness and work of fracture for the phases in the Al_2_O_3_/7wt%BaTiO_3_-platelet composite as determined by using the nano-indentation technique.

	Al_2_O_3_	BaAl_13.2_O_20.8_	Ba_4_(Ti_0.833_Al_0.167_)_12_O_27_	BaTiO_3_
**Elastic modulus/GPa**	411±13	286±11	253±3	180±11
**Hardness/GPa**	19.2±2.7	16.8±0.4	14.5±0.8	9.7±0.7
**W_total_/nJ**	5.8±0.2	6.9±0.4	7.5±0.2	10.3±0.5

The values in Table 1 demonstrate that the hardness of each phase follows the order as Al_2_O_3_ > BaAl_13.2_O_20.8_ > Ba_4_(Ti_0.833_Al_0.167_)_12_O_27_ > BaTiO_3_. A previous study also indicated that the strength of BaTiO_3_ is lower than that of Al_2_O_3_ [10]. Different from the previous studies on SiC-platelet toughened Al_2_O_3_ [2,3,4], the platelets used in the present study are much weaker than the matrix. Furthermore, a dense reaction phase is formed at the interface. However, the work of fracture (*W_total_*) as calculated from the area under the stress-strain curve during nano-indentation shows a different trend with that of hardness. From the Table, it suggests that more energy is consumed during the fracturing of the Ba-containing phases. The high fracture energy results from the possible formation of many microcracks. 

Table 2 shows the residual stress in the alumina phase of composites. A very small residual stress is present in the monolithic alumina specimen. Such residual stress may be induced by the surface grinding process. As the platelets were added to form the Ba-containing phases, tensile residual stresses were found in the alumina matrix. It is due to that the thermal expansion coefficient of the Ba-containing phases (11 ppm/K for BaTiO_3_) [11] is higher than that of alumina (8 ppm/K). Compressive hoop stresses and tensile radial stresses are expected to form at the interface. The crack could deflect around the interface. The nano-indentation analysis demonstrates that the toughening agent used in the present study is softer than that of the matrix. The crack is thus attracted to the platelets because they are elastically softer than the alumina matrix. 

**Table 2:** Residual stress and strength of Al_2_O_3_/BaTiO_3_-platelet composites. The elastic modulus and Poisson’s ratio used to calculate the residual stress are also shown.

	Monolithic Al_2_O_3_	3 wt%BaTiO_3_ -platelet	7 wt%BaTiO_3_ -platelet	14 wt%BaTiO_3_ -platelet
**Residual stress^a^/MPa**	-14*	25*	36*	73*
**Elastic modulus^b^/GPa**	400	355	257	231
**Poisson's ratio^b^**	0.24	0.27	0.14	0.15
**Flexural strength^c^/MPa**	378 ± 19	185 ± 14	161 ± 19	87 ± 7

^a^ note: determined by X-ray sin^2^ψ technique. ^b^ note: determined by ultrasonic technique. ^c^ note: determined by 4-point bending technique. * note: “-“ denotes compressive stress.

**Figure 4:**
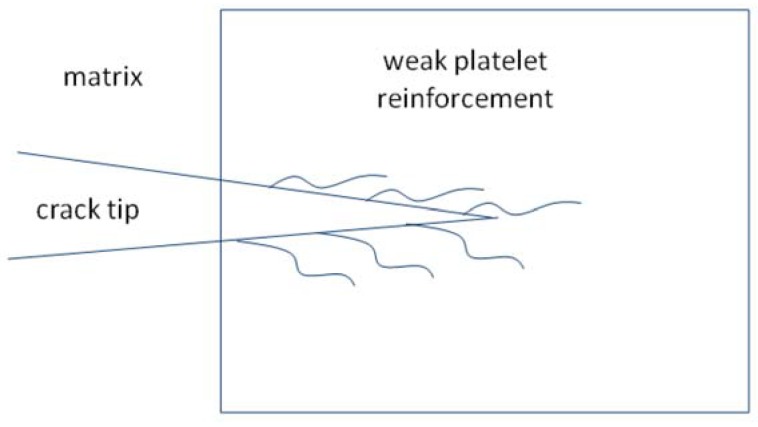
Interactions between a major crack and a weak platelet.

The interactions between crack and platelet are demonstrated in Figure 4. As a major crack penetrates into the dense BaAl_13.2_O_20.8_ interphase, many crack branches are formed to consume the fracture energy. The crack resistance of the composite is expected to be high. A schematic to demonstrate the toughening behavior is shown in Figure 4. 

The flexural strength of the composites decreases with the increase of platelet content, Table 3. It is mainly due to that the size of the platelet is far too large (length > 500 μm). The presence of such large second phase acts as a stress concentrator, or flaw, to the composites. The strength of the composite is therefore lower than that of monolithic alumina. 

**Table 3:** Anisotropic toughening effect near a platelet.

	Crack in monolithic Al_2_O_3_	Crack moves away from platelet	Crack moves toward platelet
**Crack length/μm**	190±10	275±25	127±20
**Fracture toughness^a^/MPam^0.5^**	4.8±0.3	3.1±0.2	7.8±0.5

^a^ note: determined by indentation technique.

The work of fracture for the weak materials in the platelet is higher than that of the matrix, Table 1, the toughness of the platelet composite is expected to be higher. However, as the single edge notched beam (SENB) technique was used to determine the global toughness of the composites. The toughness is not meaningful due to that the crack induced from the notch seldom interacts with any platelet. It is mainly due to that the size of multilayer platelet used in the present study is large and the number of platelets is low. It is thus difficult to determine the global toughness of the composites by using the SENB technique. The Vickers indentation technique is thus employed instead to determine the localized toughening behaviour. Each indentation introduces 4 cracks at the tips of the indentation (see Figure 3). By placing the indentation close to the multilayer platelet, the interactions between crack and platelet can then be investigated. By using the crack length induced by Vickers indentation and Eq.(2), the toughness anisotropy is observed. Table 3 shows the toughness by using the crack length induced by Vickers indentation. The toughness as calculated from the crack toward the BaTiO_3_ platelet is much higher than that calculated from the crack moves away from the platelet. It demonstrates that the addition of BaTiO_3_ platelets do consume more fracture energy. The platelets with much smaller size should be available from market soon. The use of small dummy platelets as toughening agent is expecting. 

## 3. Experimental Section 

An alumina powder (TM-DAR, Taimei Chem. Co. Ltd., Tokyo, Japan) was used to prepare the ceramic matrix in the present study. A BaTiO_3_-based ceramic powder was mixed with several solvents and binders to prepare green tapes. Several tapes were laminated together and subsequently cut into small platelets. The dimensions of the green platelets were 1.28 × 0.64 × 0.41 mm. The Al_2_O_3_ powder and green platelets were dry mixed together in a PE jar for 1h. The amount of green platelets was 3, 7 and 14 wt %. The mixture was then hot-pressed in vacuum at 1,400 °C under a load of 25 MPa for 1 h (High-multi-5000, Fujidempa Kogyo Co., LTD., Japan). The dimensions of the hot-pressed specimens were 50 mm in diameter and roughly 4.5 mm in thickness. 

The composite phase was characterized with a synchrotron X-ray source (Beam-line BL-17B1, National Synchrotron Radiation Research Center, Hsinchu, Taiwan). The diffraction angle (2θ) varied from 20° to 50°. The microstructure was observed with SEM (Philips XL-30, Netherlands). Artificial cracks were generated by Vickers hardness tester (AKASHI AVK-A, Japan) under a load of 98N. A nano-indenter (UNAT^®^, ASMEC, Germany) was also used in the present study to determine the elastic modulus, hardness and work-of-fracture of each phase in the composite. The tip was a Berkovich type nano-indenter. The load applied was 50 mN. 

The residual stress of the composite was measured by an X-ray diffractometer (Siemens D-5000, Germany) using the sin^2^ψ method [12]. The residual stress was calculated based on the displacement of plane spacing for different orientations of X-ray beams relative to the specimen. In the present study, several orientations including 0°, 18.43°, 26.56°, 33.21°, 39.23° and 45° were chosen and the residual stress was calculated by using the following equation [13] as:

(1) $$\sigma =[\frac{E}{\left(1+v\right)}](\frac{\partial \varepsilon }{\partial (\sin^{2}\psi )})$$

where *E* is the Young’s modulus, *υ* is the Poisson’s ratio and ε is the lattice strain of the material. The values of *E* and *υ* were measured by the ultrasonic reflection method. The (4 1 6) plane of the Al_2_O_3_ matrix was chosen to measure the residual stress due to its high diffraction angle and high diffraction intensity. 

For the strength measurements, the hot-pressed discs were cut into rectangular bars with a diamond saw. The rectangular specimens were ground longitudinally with a 44 μm grit resin-bonded diamond wheel at cutting depths of 5 μm/pass. The final dimensions of the testing bars were 4 × 3x45mm. The 4-point bending technique was used to determine the flexural strength of composites, respectively. The indentation technique was also used to determine the toughness anisotropy. The following equation was used to calculate the toughness, *K_IC_*, from one crack length as [14]:

(2) $$K_{IC}\phi /H\sqrt{a}=0.15k{\left(c/a\right)}^{\frac{3}{2}}$$

where φ, *k* are respectively constant of 3 and 3.2, *H* the hardness, *a* half the indent impression length, *c* the length of one crack. 

## 4. Conclusions 

The feasibility of using weak platelets as toughening agent for brittle alumina was evaluated in the present study. The platelets are prepared by using the techniques employed for the manufacture of multilayer components. Though the platelets are soft and reactive with the matrix, the major cracks can be attracted by the platelets. The crack is then stopped within the platelet by forming many minor cracks.
